# Analyzing Land Use/Land Cover Changes Using Google Earth Engine and Random Forest Algorithm and Their Implications to the Management of Land Degradation in the Upper Tekeze Basin, Ethiopia

**DOI:** 10.1155/2024/3937558

**Published:** 2024-07-30

**Authors:** Alemu Eshetu Fentaw, Assefa Abegaz

**Affiliations:** ^1^ Department of Geography and Environmental Studies Addis Ababa University, Addis Ababa, Ethiopia; ^2^ Department of Geography and Environmental Studies Woldia University, Woldia, Ethiopia

## Abstract

Land use and land cover change (LULCC) without appropriate management practices has been identified as a major factor contributing to land degradation, with significant impacts on ecosystem services and climate change and hence on human livelihoods. Therefore, up-to-date and accurate LULCC data and maps at different spatial scales are significant for regular monitoring of existing ecosystems, proper planning of natural resource management, and promotion of sustainable regional development. This study investigates the temporal and spatial dynamics of land use land cover (LULC) changes over 31 years (1990–2021) in the upper Tekeze River basin, Ethiopia, utilizing advanced remote sensing techniques such as Google Earth Engine (GEE) and the Random Forest (RF) algorithm. Landsat surface reflectance images from Landsat Thematic Mapper (TM) (1990, 2000, and 2010) and Landsat 8 Operational land imager (OLI) sensors (2021) were used. Besides, auxiliary data were utilized to improve the classification of LULC classes. LULC was classified using the Random Forest (RF) classification algorithm in the Google Earth Engine (GEE). The OpenLand *R* package was used to map the LULC transition and intensity of changes across the study period. Despite the complexity of the topographic and climatic features of the study area, the RF algorithm achieved high accuracy with 0.83 and 0.75 overall accuracy and Kappa values, respectively. The LULC change results from 1990 to 2021 showed that forest, bushland, shrubland, and bareland decreased by 12.2, 24.8, 1.2, and 15.4%, respectively. Bareland has changed to farmland, settlement, and dry riverbed and stream channels. Expansion of dry stream channels and sandy land surfaces has been observed from 1990 to 2021. Bushland has shown an increment by 17.2% from 1900 to 2010 but decreased by 19.5% from 2010 to 2021. Throughout the study period, water, farmland, dry stream channels and riverbeds, and urban settlements showed positive net gains of 484, 8.7, 82, and 26778.5%, respectively. However, forest, bush, shrub, and bareland experienced 12.17, 24.8, 1.2, and 15.37% losses. The observed changes showed the existing land degradation and the future vulnerability of the basin which would serve as an evidence to mitigate land degradation by avoiding the future conversion of forest, bushland, and shrubland to farmland, on the one hand, and by scaling up sustainable farmland management, and afforestation practices on degraded and vulnerable areas, on the other hand.

## 1. Introduction

Land use and land cover change (LULCC) without appropriate management practices has been identified as a major factor contributing to global land degradation, with significant impacts on ecosystem services and climate change, which in turn affect human vulnerability [1, 2]. The term LULCC expresses modification of the earth's terrestrial surface, mainly the results of interaction between natural and anthropogenic processes [3, 4]. It affects earth's system functioning [1, 5, 6] and its capacity to support human needs and increases the vulnerability of places and people to climatic and economic shocks [6, 7]. At the local scale, changes in the use of land and its cover affect watershed runoff, microclimatic resources, groundwater, land degradation processes, and landscape-level biodiversity [6, 8, 9]. Climate-driven land-cover modifications and human activities mediated by institutional factors, markets, policies, and global forces drive land-cover changes [10, 11]. In general, LULCC is a critical driver of global environmental change, significantly impacting ecosystems, climate dynamics, and human vulnerability. Understanding these changes is essential for developing effective land management and conservation strategies.

Therefore, up-to-date and accurate LULC maps at different spatial scales are significant for the regular monitoring of existing ecosystems, proper planning of natural resource management, and promotion of sustainable regional development [12–14]. LULCC has been observed across the world [6, 15–17]. Many studies have also been conducted at the continental [13], regional [18, 19], and local levels [6, 9, 20–31]. Many researchers have studied LULC changes in different parts of Ethiopia using GIS and remote-sensing techniques [4, 9, 20–31]. These local studies have yielded mixed results. An earlier study by Zeleke and Hurni [32] reported expanding cultivated land at the expense of forest and grass lands from 1957 to 1995 in Dembecha areas. Bewket and Abebe [9] found a reduction of natural vegetation cover and expansion of open grassland, cultivated areas, and settlements from 1957 to 2001 in the upper Blue Nile basin. In the same basin, Gashaw et al. [23] found an increase in cultivated land and built-up area at the expense of forests, shrubland, and grassland from 1985 to 2015. Mekonnen et al. [33] reported an increment in agriculture and settlement areas along with shrinks in forests and woodland cover in the Central Rift Valley of Ethiopia. In the Awash and Afar-Danakil basins, Ayele et al. [22] found the conversion of grazing land into plantation trees and area closure. In the Gelan watershed, Birhan Asmame and Assefa Abegaz [30] found a decrease in forest and wetland and an increase in shrubland, cultivated land, grassland, bareland, and settlement area. Most of these studies concluded that, like the trends on the global scale, the most remarkable change in Ethiopia is towards agricultural land, built-up/settlement areas [3, 34, 35]. Nevertheless, few other studies have shown forest recovery under protected areas. For example, Alemayehu et al. [36] found a considerable increase in dense forest in semiarid Eastern Tigray from 1965 to 2005. Similarly, Nyssen et al. [37] reported an increase in forest cover in the Bella-Welleh watershed in the Waghimra zone of Ethiopia.

Geographically, most of the studies in Ethiopia are concentrated in the Rift valley [3, 38], Blue Nile basin [9, 32, 35, 39–41], central Ethiopia, and the Awash basin [4, 21, 42]. However, such types of studies are lacking in the northwestern and eastern parts of the country [34] generally, and in the arid and semiarid catchments of the upper Tekeze basin in the Waghimra administrative zone, in particular, except for a few studies on the Tekeza catchments in Tigray [36, 43, 44]. The upper Tekeze basin is the most degraded and drought-prone area with erratic rainfall. Due to their nature of rigid topography, most of the land is either degraded or highly vulnerable to feature degradation. Due to the severity of land degradation, government intervention in soil and water conservation has been introduced decades ago. The ecological restoration programs under sustainable land management (SLM) practices, such as terraces and bunds, and establishing exclosures on communal grazing lands are among others. Following this program from 2010 to 2015, about 15 million people have contributed unpaid labour each year and more than 12 million hectares of land have been rehabilitated through implementing physical and biological conservation measures [45, 46]. However, as the level and cause of the previous degradation, drivers of degradation and LULCC and the effectiveness of conservation practices are contextual; the current understanding of LULCC and its implication to current and future degradation is not well studied. Therefore, this study was conducted in the data scarce region of Northern Ethiopia and can fill the gap by providing up-to-date LULC information and its implications for land degradation management.

Methodologically, several studies have improved land-cover change measurements in recent decades, and scientists' ability to monitor changes in LULC has improved due to the application of GIS and remote-sensing technology [1, 6]. However, accurate LULCC assessment is still a vital study for evidence-based policy advising and strategies to achieve future conservation measures [3, 8, 22, 47]. In contrast to traditional remote-sensing methods, Google Earth Engine (GEE) offers cutting-edge technologies and unrestricted access to a broader range of remote sensing data, fostering the formation of transformative land-change research issues [48]. GEE has been used for a variety of applications and at various scales of analysis [49], including drought assessment [50, 51], normalize difference vegetation index (NDVI) mapping [50–53], and land cover classification [52, 53]. Researchers have used this platform for land cover classification [52–57], and it has been suggested that GEE is very useful in spatial data management because of its fast and easy computation.

GEE also provides various pixel-based machine learning classification algorithms. Random forest (RF) support vector machines, K-nearest neighbour, artificial neural network, and classification and regression tree can improve classification accuracy by reducing collinearity and overfitting [53]. Among these, support vector machine (SVM) and RF are two widely used algorithms [56, 58] for a variety of earth science applications, including modelling forest cover, LULC, and object-oriented mapping [59–61]. However, RF has gained great popularity and become one of the best classification algorithms in LULC mapping due to its better efficiency and higher accuracy and the need for a few parameters [58]. While several studies of developed regions used RF on the GEE platform for LULC change mapping, fewer studies have used it for less developed regions [62]. Although several studies have been conducted on LULCC in Ethiopia, none of them used GEE and RF for data access and classification. Unlike previous LULCC studies that use ArcGIS and maximum likelihood classification, this study uses GEE for data access, preparation, and computation. Moreover, the RF algorithm has been used for the classification of LULC.

Therefore, the overall objective of the study was to analyse the temporal and spatial dynamics of LULC in the last 31 years (1990–2021] and its implications for sustainable land management practices in the upper basin of the Tekeze River in the Waghimra Administrative Zone of Ethiopia.

## 2. Data and Methods

### 2.1. Study Area

**Figure 1:**
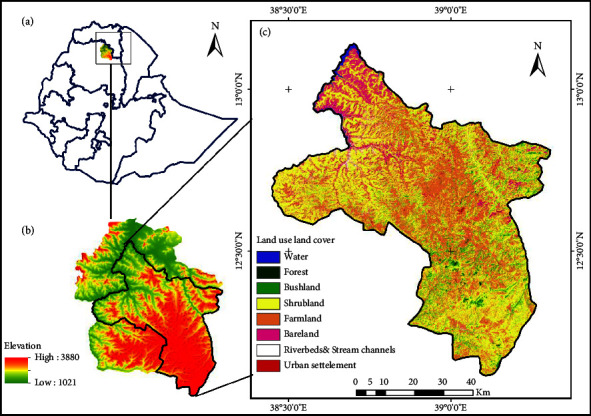
Location of the study area: (a) in Ethiopia, (b) in Waghimra Administrative Zone, (c) studied sub-basin showing land use land cover (reproduced from Eshetu & Abegaz, 2024 [63]).

The study was conducted in the upper Tekeze basin in the Waghimra administrative zone of Amhara Regional State, Northern Ethiopia. It covers an area of 4593 km^2^ and geographically extends from 12.11° to 13.13°N latitude and 38.40° to 39.30°E longitude (Figure 1). The area's elevation ranges from 1060 to 3880 m above sea level (Figure 1). The slope of the area ranges from flat plains to steep escarpments. The mean slope of the zone is 19.17%. Undulated topography with poor vegetation cover characterizes most of the Waghimra administrative zone, particularly the south west and the northern part of the study area.

This zone is one of the most drought-affected areas in the past. Land degradation, frequent drought, and the high population densities characterize it [64, 65]. Migration for short-term employment has been a feature of the livelihoods of the poorest in Waghimra. Most young people consider migration for work to be a temporary response to an inadequate cash flow [66]. Waghimra is part of Ethiopia's northern highlands, which are highly vulnerable to climate change, including extreme climate events such as droughts. High vulnerability to drought and famine, growing population pressure, and land degradation have resulted in reduced yields. People residing in Waghimra are frequently in immediate need of food aid. Although NGOs and governmental organizations could encounter basic needs, the root cause of food insecurity is related to the region's degraded ecosystem and not yet tackled.

**Figure 2:**
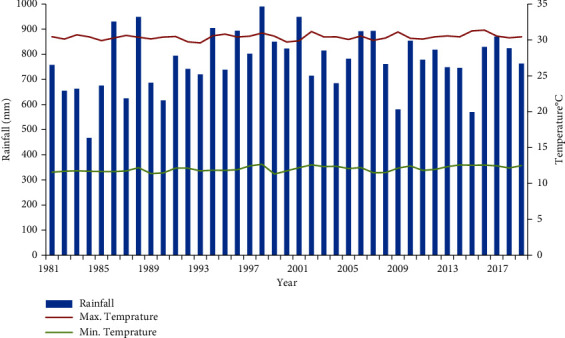
Annual rainfall and mean annual minimum and maximum temperature of the study area from 1981 to 2021 (derived from monthly CHIRPS-v.2) (reproduced from Eshetu & Abegaz, 2024 [63]).

In almost all parts of the Tekeze basin, the summer (*Kiremt*) rain starts in June and ceases around the end of August [67]. Specifically, the mid- and high-altitude areas receive rainfall from late June to early September, while in the lowland parts of the basin, it extends from early July to mid of August. The primary crop production system is rain fed during the summer season. About 63% of the annual rainfall occurs in July and August. The area's annual minimum and maximum temperatures are 12.02°C and 30.4°C, respectively (Figure 2).

### 2.2. Data

This study used level 2 surface reflectance (SR) products from Landsat 5-TM and landsat 8 OLI sensors available in the GEE. The U.S. Geological Survey (USGS) provides the level 2 SR data from Landsat 8 generated using the Land Surface Reflectance Code (LaSRC) algorithm. For Landsat 4 to 7, SRs are derived with the Landsat Ecosystem Disturbance Adaptive Processing System (LEDAPS) algorithm [68]. Landsat collection 2 level 2 data have undergone a series of atmospheric and geometric corrections and are science products with values added and highly processed Landsat products [69, 70]. These SR collections ensure that multidate images are comparable and allow more stable and reliable land change analyses [52]. SR is generally more appropriate for measuring and monitoring vegetation and other land cover types at the land surface [69, 71, 72].

Four sets of digital satellite imagery from TM and OLI sensors for the years 1990, 2000, 2010, and 2021 were used to examine LULC dynamics (Table 1). Years of analysis (1990, 2000, 2010, and 2021) were selected purposely to align with significant sociopolitical events in Ethiopia and Northern Ethiopia. Accordingly, the 1990 image indicates the land and environmental conditions during the Derg regime. The year 2000 represents the aftermath of the fall of the Derge regime and the early period of Ethiopian People Revolutionary Democratic Front (EPRDF) regime. During these periods, environmental management and protection were not the government's top priorities. The year 2010 represents the efforts of soil and water conservation (SWC) and SLM programs in different parts of Ethiopia, including the study area. In 2010, the government of Ethiopia launched nationwide ecological restoration and area exclosure programs [73, 74]. Finally, the 2021 image represents the current biophysical status and observed changes after 2010 area exclosure and degraded land restoration policy. In order to minimize the seasonality effects, images were mainly selected from January to March.

#### 2.2.1. Auxiliary Data

Previous studies have shown that different remote sensing indices are sensitive to different types of LULC and enhance classification accuracy [54, 55, 57, 75, 76]. To improve classification accuracy, spectral indices retrieved from the original bands, such as NDVI, Normalized Difference Water Index (NDWI), Normalized Difference Moisture Index (NDMI), and Normalized Difference Built-Up Index (NDBI), were used as additional bands in this study. Furthermore, the tasselled cap ratio was derived and incorporated in the classification bands. The tasselled cap component is widely applied to characterize vegetation conditions. These indices measure the presence and density of green vegetation, total reflectance, and soil moisture content [57]. After visually inspecting the pattern and accurateness of the index, by overlaying on known sample points, the derived indices were evaluated and added to the classification bands. In addition, topographic variables, such as elevation and slope, were used as auxiliary variables for the classification.

### 2.3. Data Processing and Classification Overview

**Figure 3:**
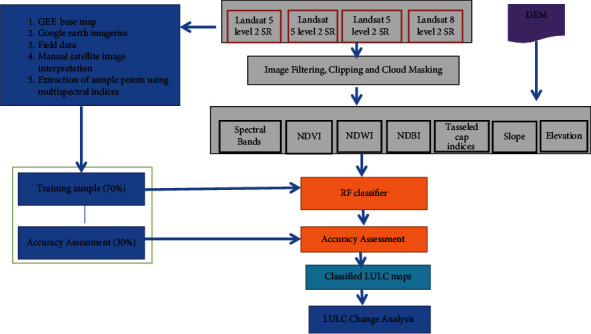
Schematic overview of the methodological framework of the study.

An overview of the methodological framework applied in this study is shown in Figure 3. The majority of the image processing and analysis for the study was implemented through GEE. All data sets used in this study are freely available on GEE. We have filtered cloud-free, dry season (January to February) SR images clipped to the study area. Indices were also derived from these cloud-free images and added as an independent band to the original Landsat images. Indices and other axillary data were generated using the GEE API. After we had obtained all the required bands, we overlaid the training and validation points of each LULC on the final image. The sample points were selected using field data, Google Earth imageries, and NDVI thresholds. Then, tuning machine learning classifier hyperparameters, generating classification maps, and assessing the accuracies of classified maps were performed. Finally, the total area and the change of each LULC class and the temporal pixel transition matrix from one LULC category to the other were calculated to assess and analyse LULCC patterns.

### 2.4. LULC Classification Schemes

As described in Table 2, eight LULC types were identified in the study area. All training and validation samples were collected based on field survey data and manual visual interpretation of high-resolution images from Google Earth and the GEE base map. This method is widely applied in the literature [53, 55, 54]. Multitemporal Google Earth aerial imageries were used to select suitable training sites for the eight LULC types from the acquired Landsat images. For the earlier study periods (1990 and 2000), the available Google Earth imageries corresponding to the study area are less precise than those from 2010 to 2021. However, the false colour interpretation of the acquired satellite images was employed to identify sample points from all land covers. This photo interpretation technique was also enhanced by incorporating axillary data in the classification algorithm. For example, the NDVI threshold was used to identify vegetation. Dry and high-reflecting bareland was also easily identified with the help of the tasselled caped dryness index. In addition, the temporal stability principle was considered to select sample points. For example, church forests are among the convenient sample points in the study area due to their stability for extended periods. Riverbeds and urban settlements were also easily identifiable features and have been stable since their existence. A total of 1721 ground control points (GCP) were collected and used for the LULC classification.

### 2.5. Classification Method and Process in GEE Using RF Algorithm

Machine learning-based classifiers help identify complex patterns while at the same time minimizing the problem of data dimensionality [53]. The RF consists of many individual decision trees. Each decision tree has several nodes, and the majority vote determines the result. The algorithm not only randomly selects subsamples from the input variables but also randomly selects the best feature through a voting process to establish the splits in the nodes of trees. According to a systematic review from 2010 to 2019, the RF algorithm is one of the most frequently used classification algorithms. Hence, this study applied the RF classification algorithm for LULC classification. Among the 1721 sample points, 70% were used for training the RF classifier, and the remaining 30% were used for validation. The number of decision trees was set to 50, which was found to be the optimum number producing a good accuracy level. The accuracy evaluation was performed using indices including the user's accuracy (UA), producer's accuracy (PA), overall accuracy (OA), and kappa coefficient [77–79]. These accuracy indices are calculated by constructing a confusion matrix using the GEE syntax. The GEE inbuilt code for construction of error matrix and calculation of LULC accuracy is based on Stehman [80].

## 3. Results and Discussion

### 3.1. Accuracy Assessment of the LULC Maps

**Figure 4:**
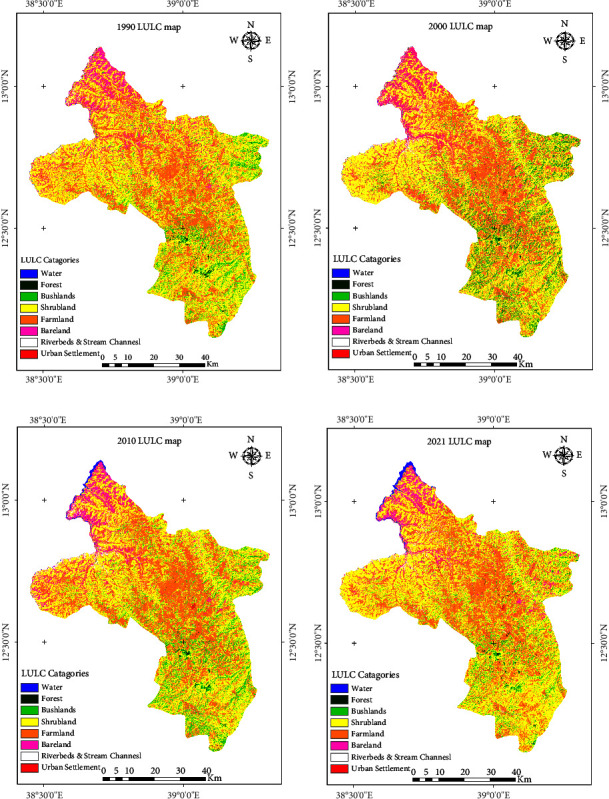
LULC of the arid and semiarid land of the upper Tekeze basin in 1990 (a), 2000 (b), 2010 (c), and 2021 (d).

After classifying the images using the collected samples, accuracy was assessed.  Table 3 shows the details of the accuracy evaluation of the RF classification (user's accuracy (UA), producer's accuracy (PA), and overall accuracy (OA)) and kappa classification. The accuracy evaluation for each classified image was calculated by constructing a confusion matrix. The RF classifier produced good overall accuracies, with overall accuracy assessments of 0.83, 0.86, 0.88, and 0.88 for 1990, 2000, 2010, and 202, respectively, and the kappa accuracies were 0.75 (1990), 0.79 (2000), 0.83 (2010), and 0.82 (2021). Water had high classification accuracies each year because its reflectance is easier to distinguish from other categories, whereas bareland and urban settlements had low accuracies. It was also a challenge to differentiate the spectral characteristics of bushland and forest areas due to the narrow spectral signature difference among these land cover types. Although the area coverage of forest was low in the study area, it was essential not to merge with bushlands. We think it is crucial to quantify and identify the church forests, riverine trees, and plantations that are sparsely located across the study area. Compared with surface water and vegetation, the built-up and barren areas showed a relatively low accuracy. The aridity of the northern part of the study area challenged the identification of barren land from urban settlements, dry stream channels, and sandy flooded and sparse shrubs. However, the incorporation of the axillary data, elevation, slope, and tasselled cap indices, helped improve the identification of these resembling spectral characteristics. Note that urban settlement in 1900 was almost absent because it was very small during this period, and most of the settlement was a rural settlement with a similar reflectance value with farmland and bareland.  Figure 4 shows the land cover classification maps for 1990, 2000, 2010, and 2021.

### 3.2. LULC Change in the Study Area

In 1990, shrubs covered 55.93% (256866 ha) followed by agriculture, which covered 26.84% (123250 ha), and bareland, which covered 9.11% (39408 ha) of the study area (Figure 4(a) and Table 4). Water bodies, forests, and bushlands each accounted for 0.1, 0.5, and 7.05% of the study area, respectively. Urban settlement and residential areas covered only 1.66 ha of the study area. In 2000, shrubland and farmland remained the most predominant land cover types, accounting for 242284 ha (52.76%) and (132724.28 ha) (28.90%) of the study area, respectively (Figure 4(b) and Table 4). Forest cover increased by 160 ha, accounting for 0.51% of the entire study area. In 2000, bushland was 42334 ha, increased by 30.7% (9953 ha) from the 1990s coverage. Bareland and stream channels combined covered 8.45% of the study area in the 2000 LULC map.

On 2010 LULC map, the study area was covered by 51.30% shrubland, 30.04% farmland, and 8.33% bushland (Figure 4(c) and Table 4). Other land use types covered the remaining 10.28% of the area. Shrubland, bushland, and forest cover decreased by 6691, 4083, and 406 ha, respectively, from 2000 to 2010. These reductions were due to the expansion of 5212 ha farmland, 14 ha bareland, and 2364 ha riverbed and stream channels. Compared to the preceding study period, the water body has increased dramatically by 1529 ha. This was due to the accumulation of water in the Tekeze River's channels due to the Tekeze Hydroelectric Dam's construction. Forest cover has decreased by 4066 ha compared with the previous study period. Except for what has been observed in bushland decline and shrubland increase, LULC distribution in 2021 is comparable with that in 2010. The study area was still dominated by shrubland, followed by farmland, as they have been for the last two decades. Only water bodies, shrublands, and urban settlements expanded. Unlike the past 20 years of the study period, bush lands have experienced the most significant decline in this period. Conversion to shrubland and encroachment of riverbeds and flooded sandy stream channels are the prime causes of bushland decline.

The general trend in the last three decades (1990–2021) showed that water, farmland, stream and riverbed, and urban settlements and residential areas were increasing. The remaining land covered classes, forest, bush land, shrubland, and bareland, decreased by 12.17, 24.79, 1.18, and 15.37%, respectively. Settlement, water bodies, riverbed, and stream channels have also expanded, indicating the expansion of dry stream channels and sandy land surfaces in the study area. Generally, the water body has increased by 81.17 ha every year from 1900 to 2021. In contrast, forest land increased by 12.17% from 1990 to 2021, with a rate of 8.84 ha yearly. Bushland has shown a continuous increment in the first two consecutive decades (1900–2010) by about 17.24%. However, it experienced a decline of 19.56% in the next decade from 2010 to 2021. Throughout the study period, water, farmland, stream channels, riverbed, and urban settlements showed positive net gains of 483.97, 8.70, 82.14, and 26778.47%, respectively. At the same time, forest, bush, shrub, and bare LULC classes experienced losses of 12.17, 24.79, 1.18, and 15.37%, respectively.

**Figure 5:**
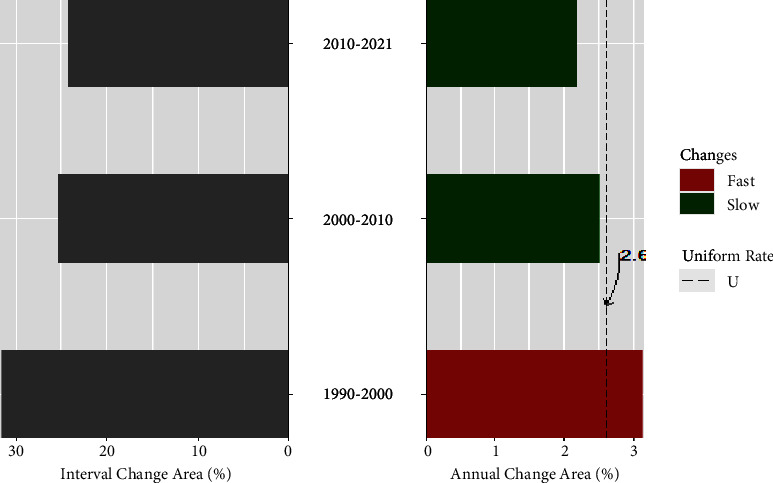
LULC change intensity showing the size and speed of change across the three studied time intervals (1990–2000, 2000–2010, 2010–2021). Bars that extend to the left of zero show the percentage of change during the corresponding interval, and bars that extend to the right of zero show the percentage of change per year within each time interval. Uniform rate (U) assumes if the annual changes were spread evenly over the study period.

Figure 5 shows that the rate of LULC change is not stationary during the studied time intervals. The length of bars on the right indicates that the annual change in the three periods was not identical. Left of Figure 5 shows that the size of the change during the first-time interval (1990–2000) was the largest. The next two periods experienced decreasing change. On the right side of Figure 5, the first interval (1990 to 2000) was with the fastest change intensity in terms of the annual rate of LULC change, and it decreased in the period between 2000 and 2010 and then in the latest period.

### 3.3. LULC Transition Dynamics

**Figure 6:**
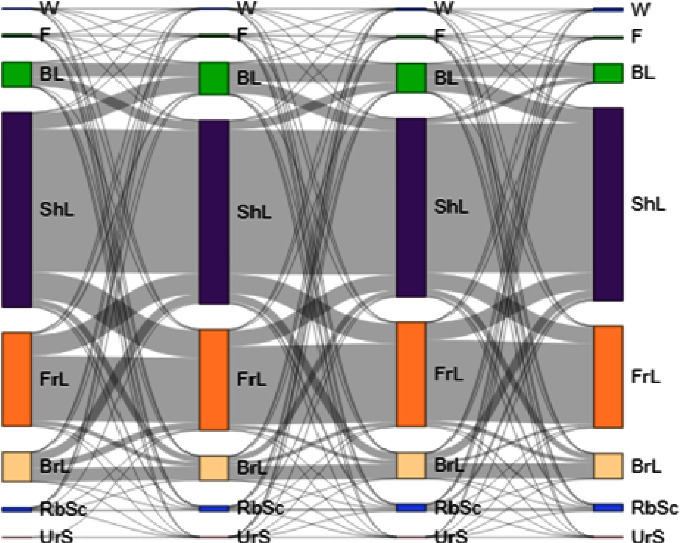
Multistep LULC transition between 1990 and 2021 in the upper basin of the Tekeze River; size of colored bars and transition links are displayed proportionately to area in km^2^ (note: W = water body, F = forest, BL = bushland, Shl = shrubland, FrL = farmland, BrL = bareland, RbSc = dry river beds and stream channels, and UrS = urban settlements).

**Figure 7:**
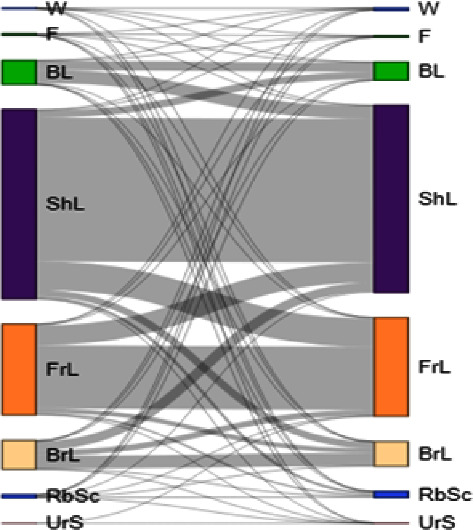
One-step LULC transition from 1990 to 2021 in the upper Tekeze Basin. The left bars represent LULC categories and their proportion in 1990; the right side bars represent the LULC categories and their proportional size in 2021. Links are displayed proportionately to area in km^2^ (note: W = water body, F = forest, BL = bushland, Shl = shrubland, FrL = farmland, BrL = bareland, RbSc = dry river beds and stream channels, and UrS = urban settlements).

The land-use transition matrix between 1990 and 2000, 2000–2010, and 2010–2021 was derived using JavaScript code in the GEE environment. Multistep (Figure 6) and one step (Figure 7) transition graphs were produced in the OpenLand package in *R* software [81]. The purpose of generating a transition matrix was to identify the magnitude of the transition of pixels under a specific land cover class to another land cover category. This matrix shows the magnitude and direction of LULC changes within the study area. All LULC types experienced changes, and the intensity of change differed among the seven LULC classes.

#### 3.3.1. LULC Transition from 1990 to 2000

Multistep LULCC transitions from 1990 to 2000, 2000 to 2010, and 2010 to 2021 are presented in Figure 6. About 60.5% of land covered by water in 1900 transformed to 2000 without change. The remaining portion of land covered by water in 1990 (147.37 ha or 29.2%) was changed to dry riverbeds and stream channels. Despite the conversion of water bodies to dry riverbeds and stream channels in 2000, 67.3 ha of land covered by a dry riverbed in 1900 also changed to a water body in 2000. Nevertheless, from 1990 to 2000, other land use types also changed to a water body. The remarkable conversion was from a shrub in which 199 ha of its cover changed to a water body. Despite the net areal extent increment of frost from 1900 to 2000, 809 and 502.42 ha of forest were changed to shrubland and bushland, respectively. However, another 613.55 and 552.61 ha of land covered by bushland and shrub in 1990 have changed to forestland. The appearance of trees and agroforestry in irrigated areas might be reasons for the addition of 345.34 ha to forests. During 1900–2000, 56.47% of bushlands remained unchanged, while 38, 3.33, and 1.9% were converted to shrubland, farmland, and forest. The remaining small portion has changed to water, bareland, stream channels, and riverbeds. From 256610 ha of land covered with shrubland in 1900, 73.78% of it remains unchanged. The other 3588, 20411, and 7885 ha of shrubland changed to farmland, bush land, and bareland, respectively. The reaming 1.5% of shrub bland changed to water, forest, riverbed, and stream channel. Bareland experienced the greatest instability; only 46% of its coverage in 1900 remained unchanged from 1990 to 2000. The primary conversion was towards shrubland, farmland, and dry riverbeds. In this transition period, an increase in the areal coverage of dry riverbed and stream channels has been observed. This increment was due to conversion from shrubland, farmland, and water bodies. This conversion of shrubs and farmland into dry riverbeds indicates severe drought that leads to dry, flooded farmlands and sandy and highly dispersed shrubs.

#### 3.3.2. LULC Transition from 2000 to 2010

Water has experienced the most tremendous increase in this period, gaining 1579.11 ha of land. While 19.23% of its cover comes from 2000, 18.41%, 27.63%, and 27.67% were transformed from shrubland, bareland, riverbeds, and stream channels. Forest cover has decreased from 2807 ha to 1935 ha due to the encroachment of shrubs and bushlands. Bush and woodland have gained a net of 3754 ha of land from 2000 to 2010. Almost 2000 ha of the increment come from shrubland. About 400 ha of land were also converted from farmland and forest. During 2000–2010, shrubland and farmland showed the highest stability, in which 79% and 78.45% of the pixels were unchanged. On the other hand, forest, bush, and bare and dry riverbeds have experienced the greatest transition in which only 40, 58.57, 59.22, and 53% of their coverage, respectively, were unchanged from 2000 to 2010. Although there was a bidirectional transition, the transition of pixels of farmland and shrubland to bareland was marked. Similarly, land that was covered by bareland and shrubland has also changed to farmland in this period. The areal coverage of forest has shown a reduction because of the conversion to bush and shrublands. Both conversions to and from stream channels and riverbed were observed during this period. The net increase in the area coverage of this land cover type was primarily due to the conversion of shrubs and barelands to stream channels and riverbeds. Unlike the previous study period, urban settlement has considerably increased in this period.

#### 3.3.3. LULC Transition from 2010 to 2021

During the final period, water bodies, shrubland, and farmland have shown greatest stability with 88.32, 83.93, and 77.19% unchanged land cover from 2010 to 2021. However, forest cover, bushland, and bareland have shown the lowest transition possibility having only 46.46 45.68, 57.9% of their 2010's coverage transformed to 2021 coverage. 19034 and 1028.07 ha of bushland have converted to shrubland and farmland in 2021, respectively. On the other hand, much of the forest cover in 2010 has changed to bushland. Shrubland, which has the highest gain in this period, has gained majority of its area coverage from the conversion of bushland, bareland, dry riverbeds, and stream channels. The water accumulation on the Tekeze river channel from the end of 2009 continues to expand up to 2021.

**Figure 8:**
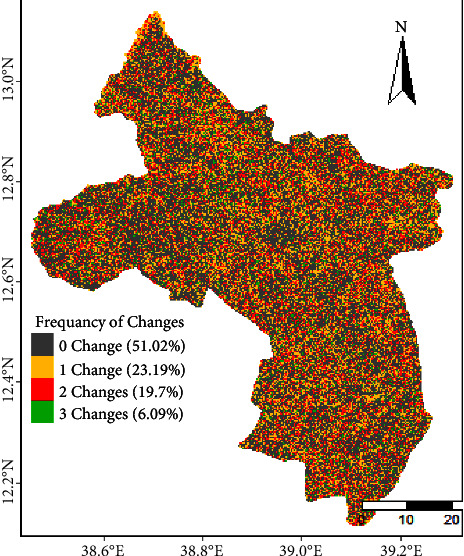
Frequency of changes of pixels from one LULC category to other categories between 1990 and 2021 at three time intervals (1990–2000, 2000–2010, and 2010–2021) in the upper Tekeze basin from.

The LULC of the upper Tekeze basin has experienced irregular gain and loss; however, high intensity of change occurred between the study periods.  Figure 8 shows the frequency of change of each pixel in the study time interval and the areal percentage of these frequencies of changes. Almost half of the land in the overall basin experienced LULC change from 1990 to 2021. 51.01% of the basin was unchanged throughout the study period. However, 23.2, 19.7, and 6.1% of the land experienced 1, 2, and 3 times change, respectively, during the study period.

### 3.4. LULC Distributions and Changes along Different Slope Classes and Agroecological Zones

**Figure 9:**
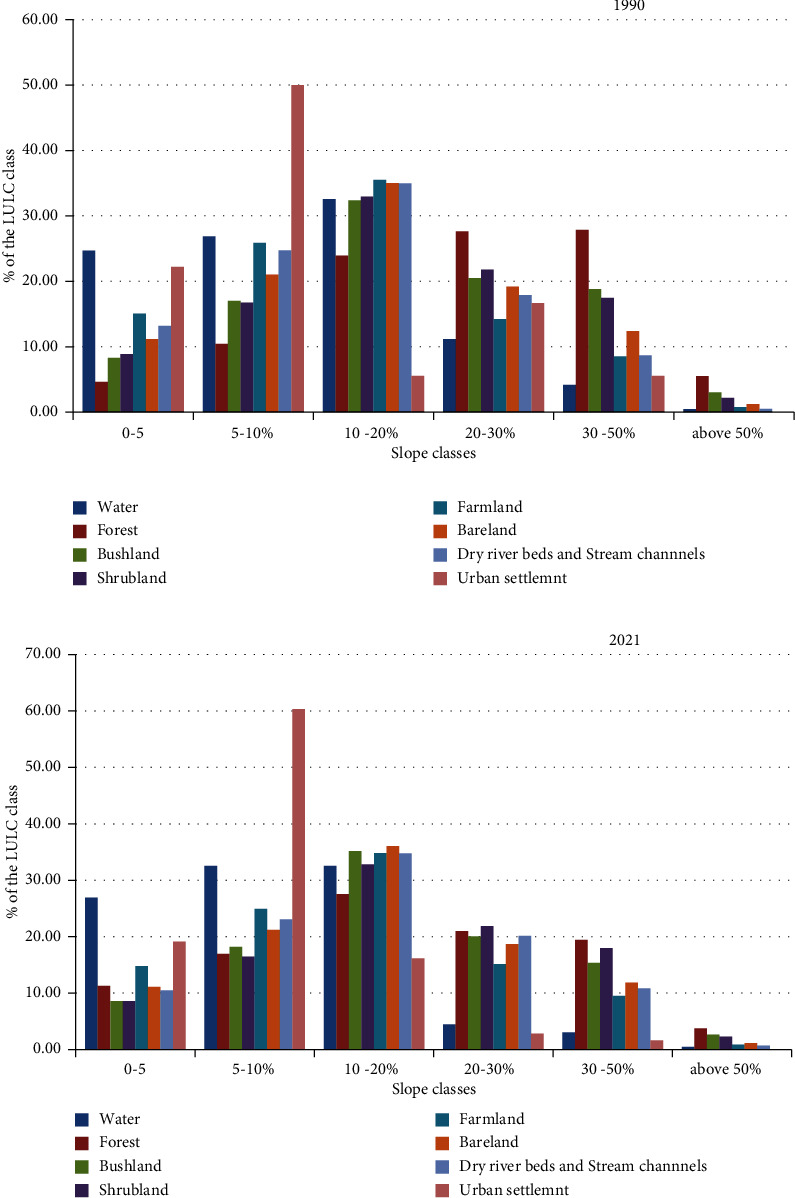
Distribution LULC types across different slope gradients in the upper Tekeze basin in 1990 (a) and 2021 (b).

In the study area in 1990, 65.54% of the land cover lay below 20% slope. In addition, 32.66% were between 20% and 50% slope gradients (Figure 9(a)). Only 1.76% of the land cover was found above the 50% slope. On average, 10.8% in 1990, 12.1 in 2000, 14.76 in 2010, and 14.48% of the total land cover lies on less than a 5% slope. Likewise, 23.64% in 1990, 29.89 in 2000, 27.61% in 2010, and 26.67% in 2021 were found between 5 and 10% slope classes. The slope gradient between 10 and 20% also holds 31.06, 28.9, 29.45, and 30.76% of the total land cover maps in 1900, 2000, 2010, and 2021. In all study periods, more than 26% of the lands cover lies between 20 and 50% slope gradient. A very small proportion of the total land cover, less than (1.76%) was found above the 50% slope. 35% of the farmland lies between the slope classes 10–15.

Regarding the distribution of the different LULC categories across the different slope gradients, Figure 9 shows the percentage of the total land cover of each class that lies on each slope class. Looking at the recent LULC distribution among different slope classes, the forest was distributed throughout all slope gradients. Almost all the gentle and higher slope gradients were nearly equal in forest coverage (Figure 9(b)).

Farmland was distributed across all the slope classes. Only 15% of the farmland was found below a slope of 5%. While 60% of the total farmland is found between 5 and 20% slope, there is still a significant proportion of farmland (14.47%) between 20 and 30% slope gradient. The study area has a long history of crop cultivation where most of the cultivable land is already occupied by croplands. Recent expansion of farmlands is on high slope areas where critical care is needed in order to protect soil and water conservation. A similar trend of increased cultivation of steep slopes has been observed in other parts of Ethiopia [31]. In this agroecology, the farmland lies in high slope classes. This aggravates land degradation through water erosion processes and washes away soil nutrients. The highland agroecology which most of its part is distributed across a high slope gradient and elevation constitute Dega agroecology. This agroecology is good for agricultural production. Particularly, legume plants, wheat, and barley are produced on fragmented plots. This high slope area cultivation in this zone requires continuous efforts to conserve soil and water so that the productivity will sustain in the future.

Although we have found forests, bushes, and shrubs fairly distributed from the lower to the higher slopes, their proportion is considerably high on the higher slope gradient. Forest, bush, and shrub percentages on the extremely high slop area (i.e., above 50%) are 4.4%, 2.57%, and 2.36%, respectively. This may be due to the growth of vegetation in areas with less human intervention.

Land use is the primary driver of land degradation, when compared with soil type, terrain, or climatic factors. The general pattern of LULC change shows that the study area is either degraded or vulnerable to degradation. In addition to this, the trend of LULC change indicates no sign of improvement in vegetation cover. The observed increase in farmland, stream channels, and riverbeds, conversion of bushland to shrubs and bareland, and decrease in forest cover are indications of land degradation in the study area. For instance, as farmland land was expanded at the expense of other land use and land cover units, bushland and forestland declined, increasing the vulnerable area to soil erosion. The increase in dry riverbeds and stream channels is also at the expense of shrubland. The increase in dry riverbeds and stream channels alone is an indication of degraded land. The combined effect of erratic rainfall, poor vegetation cover, and high human and livestock population pressure on land resources may have contributed to an increase in dry riverbeds and stream channels and further expansion of bareland.

From the agroecological perspective, the high and midaltitudes are better in terms of forest and bushland distribution. Bareland and dry riverbeds are less available in this agroecological zone. For example, Gazigibla district, whose major part lies at mid- and high latitudes, holds 65.2, 44.87, 54.42, and 49.8% of the total forest coverage in 1990, 2000, 2010, and 2021. The lowland part of the study area, which has a semiarid and arid climate, was found to be more vulnerable to land degradation due to its LULC status. The finding showed that this part of the agroecological zone has greater area coverage of bareland, river beds, and stream channels. Due to this and a steep slope and ridged topography, the majority of the land is vulnerable to soil erosion. For instance, the lowland part of the study area, Zquala district, has shown significant degradation as expressed in vegetation cover and expanded bareland. Riverbeds and stream channels are also abundant, which are indications of gully formation.

### 3.5. Implication of the LULC Dynamics to the Management of Land Degradation in the Study Area

**Figure 10:**
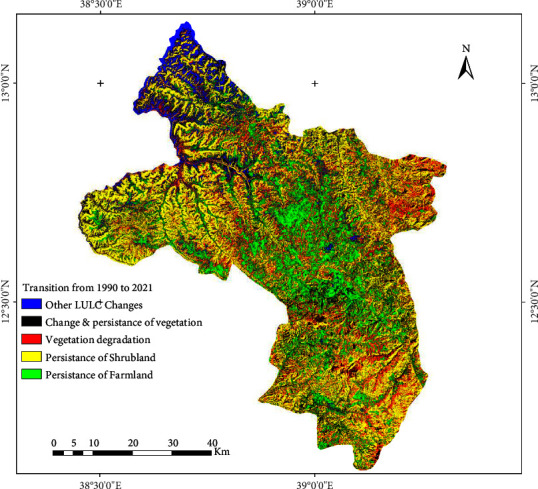
Changes/conversion to and persistence of LULC in the upper Tekeze basin from 1990 to 2021.

Land cover is one of the factors that determine the rate and status of land degradation [30]. Reduction in vegetation cover is the major cause of soil erosion particularly in mountainous ecosystems. After vegetation cover is removed, factors such as the steepness, length, and shape of a slope become important accelerators of erosion [32, 82]. This process increased sheet, rill, and gully erosion by reducing the protection of soil cover. This study identifies land use transition from and to vegetation cover to assess the degradation level and trend. The vegetation cover at the base year (1990) was minimal; particularly forest was only 0.47% of the total basin and bushland was also 7.5% of the basin in 1990. This indicates that the LULC of the study area was already degraded and was potentially exposed to soil erosion. Shrubland that covers 56% of the study area is characterised by very spare small tress and bush, and the ground consists of exposed rock and soil. The land covered by this LULC category is also prone to soil erosion. Considering the topographic and LULC diversity of the basin, dense bushes and forests are helpful for erosion management and reduction of land degradation. The study assessed the level of afforestation or reforestation and forest conservation: by studying the persistence of or change to vegetation from 1990 to 2021. Conversion of forest, bushland, and shrubland to other LULC types (urban settlements, bareland, riverbed and stream channel, and farmland) was identified as vegetation degradation, while the reverse change is gain of vegetation.  Figure 10 shows the distribution of vegetation gain and loss and persistence of farmland and shrubs from 1990 to 2021.

The overall trend observed in the study area is the decline of forest cover, bushland, and shrubs and an increase in farmland (including rural settlement), dry riverbeds and stream channels, and urban settlement. Even forestland was and is very small in area coverage; no afforestation or reforestation through plantation was observed over the study period (Figure 10). Bushland has decreased, but was increasing from 2000 to 2010 due to area closure introduction; however, it has decreased from 2010 to 2021. Bareland has changed to farmland and shrubs, washed away by flooding, and continuously changed to dry riverbeds and stream channels. These LULC types are highly susceptible to erosion. Moreover, slope, elevation, and high surface temperature aggravate the rate of soil degradation unless an urgent measure is taken. In the Ethiopian mountains, soil degradation due to water erosion is a major threat to agricultural production [82]. The observed expansion of dry stream channels is an indication and aggravating factor of soil erosion.

A land cover change interacts with the hydrological cycle. Infiltration, runoff production, and soil erosion are determined by the nature of ground cover. Low level of vegetation cover and expansion of barelands' area are responsible for high surface runoff and hence soil erosion. The observed LULC dynamic of the basin showed that there is poor vegetation cover and extensive land of barelands. Furthermore, there is increased areal extent of dry riverbeds and stream channels which are the results of high surface erosion during intense rainfall. Particularly the ridged topography of the northern part of the basin there is expansion of increased dry river stream channels. The major rivers and their tributaries form deep canyons and gorges with steep and narrow river valleys. Along the course of these valleys there are many springs that emanate along the contacts of the different rocks [83] as they draw downward to the lower part of the basin; they have developed a wide flood plain and eroded bare grounds, expanding dry river beds. Due to the low retention capacity of the soil, low vegetation cover, and steep slope, the erratic rainfall has produced wide and many irregular dry streams. These many stream channels bring flow to the farmlands on the hillside and lowland areas, destroying vegetation and crops during the rainy season.

In addition, the constructed hydroelectric dam has changed the hydrological regime of the basin by increasing water at the back of the dam as well as by widening the Tekeze river channel. The sideways of the river have been washed away, shrubs have been reduced and bare, and dry riverbeds have expanded. In the same basin, Welde and Gebremariam [82] reported that the mean annual stream flow and annual sediment yield of the Tekeze dam watershed show an increase in average annual stream flow. Similarly, sediment yield change shows an increment. Implementation of soil and water conservation measures and construction of hydropower dams and microdams for irrigation are predominantly found in the upper catchments. The land use dynamics observed in the study area would have a clear effect on the erosion rate, sediment yield, and expansion of many irregular dry riverbeds and stream channels. This has implications for the management of land degradation in the basin, requiring integrative effort to manage the land change system in the way it benefits the soil and hydrological regimes.

## 4. Discussion

The LULC change studies provide useful information for a better understanding of previous practices, current LULC patterns, and future LULC trajectory [83]. A change in LULC is one of the major causes of changes in Earth's system functioning [84]. The expansion of agricultural land, for example, occurs at the expense of shrub and other vegetated lands which finally causes soil erosion, sedimentation, and loss of biodiversity. Land cover changes are usually caused by human activity such as urbanization, agricultural expansion, and deforestation. This study shows the LULC changes dynamics in the upper basin in Tekeze in the Waghimra Zone of Northern Ethiopia for the last three decades. Advanced techniques of LULC classification aided by the GEE cloud computing platform and RF algorithm were used to study the LULC dynamics of the study area. The applied methods produced excellent overall accuracy and kappa coefficient. The overall accuracies are 0.83, 0.86, 0.88, and 0.88 with kappa coefficients of 0.75, 0.79, 0.83, and 0.82 for 1990, 2000, 2010, and 2021 classified images, respectively. The overall accuracy and kappa coefficient of this study are comparable with [12, 84].

The findings of this study show that there is a decline in vegetation cover and no marked improvement in forest cover and other vegetation as expected following the continuous land management programs. This finding is in line with [3, 40, 85–87] who reported a substantial decrease in forest cover, grasslands, and bush-shrub-woodland. However, the finding is against reports by [88] who reported an increase in woody savannas, deciduous broadleaf, grasslands, permanent wetlands, and mixed forest areas and reductions in croplands and water bodies in the Baro-Akobo River Basin of Western Ethiopia. Similarly, in recent LULC dynamics of the Blue Nile River, an increment in plantation has been observed in the North Gajjam sub-basin [41]. However, in this study, no significant increment was observed in plantations such as eucalyptus trees.

In the same basin, Nyssen et al. [37] reported an increase in vegetation cover in the Bella-Welleh watershed in the Waghimra zone. Because our study covers a large areal extent and was not limited to a single watershed, the findings are not comparable with those of this study. However, Welde and Gebremariam [89] reported a reduction of shrubland and grassland due to the expansion of the agricultural practice in the area in the same basin. In another part of Ethiopia, but with the similar environmental conditions, Shiferaw et al. [90] reported a decrease in bush-shrub-woodland and natural forests in the Afar region. In the Blue Nile basin, Gashaw et al. [91] reported decreased coverage of shrub/bush LULC for 30 years (1985–2015). Similar decreasing trends were reported by [4, 21]. The continuous decline of forest, shrub, and woodland cover was primarily due to the expansion of the urban built-up areas and cultivated and rural settlement areas. The decrease in shrub/bushland use and land cover implies that the land is vulnerable to soil erosion and flooding, affecting farmland productivity in the areas. Despite the loss of vegetation cover in the study area, the areal extent of conversion to bareland was not found to be a marked finding. Nevertheless, there was a decline in bareland cover due to conversion to farmland, riverbeds, and stream channels. Unlike other studies such as [30, 41], water body has expanded in the study area during the recent decades of the study period. Surface water accumulation has increased along the river channel of the Tekeze River, particularly at the back of the Tekeze hydroelectric power dam. This increment has positive implications for the surrounding ecosystem and livelihood of communities residing in the study area.

The pattern of temporal LULC changes was highly complex, with a multidirectional transition from one LULC to the other. It does not have a clear trend except for the overall transition towards the LULC categories that have gained from decade to decade and throughout the study period. Due to climatological effects, LULC has undergone a series of transitions, for example, from farmland to eroded/flooded bareland, from bareland to dry stream channels and riverbeds, and to flooded, sandy shrubland. The probability of transitions to forestland and bushland was very low from decade to decade. Instead, a higher probability of transitions was recorded from all land use types to farmland, shrubland, bareland, and dry riverbeds and stream channels.

All the changes observed in the study area showed that there is unhindered land degradation as manifested by the decline of the existing forest cover and no sign of afforestation. Furthermore, the degradation of bushes and shrubs, expansion of dry stream channels, and barelands are causes and indications of degradation. Many studies have shown that surface erosion is minimal in areas where the soil is covered by vegetation [4].

What is specific to this study is that the expansion of farmland is less pronounced than other studies such as Mekonnen et al. [3] and Moisa et al. [86]. Farmland has increased only by 8.70 from 1990 to 2021; there was a slow transition of other land cover types to farmland every decade. The possible reason is that almost no land was left for further expansion. Topographic, hydroclimatic, and soil degradation are challenges to farmland expansion as well as crop production. However, the recorded increase in cultivated land was at the expense of bush and shrubland. This aggravates the vulnerability of soil to erosion.

LULC driving factors in Ethiopia include demographic, socioeconomic, and institutional factors [31]. Like the rest of the Ethiopian basins, these factors apply to the upper Tekeze basin. Nevertheless, physical and climatic elements that trigger significant LULC [5] are among the driving forces in the arid and semiarid land of the upper Tekeze Basin. As observed in the LULC change across agroecologies, the drier and rigid topography of the lower basin was a hotspot of vegetation degradation and hence is susceptible to soil erosion by water. This study demonstrated that although there is not significant farmland expansion, urban and infrastructural induced LULC change in this marginal arid and semiarid basins, and topographic, agroecological, and climatic factors such as aridity and frequent drought could cause land cover change and challenge land degradation management.

Although a lot of implications can be associated and discussed based on the observed LULC change magnitude and direction, future research should focus on understanding of the impact of LULC change on the water resources, ecosystem productivity, and livelihood security in the study area.

## 5. Conclusion

This study focused on the status and trends of LULC changes in the semiarid and arid land catchment of Tekeze River in the Waghemra Administrative zone of Amhara region, Ethiopia. LULC classification was performed using the RF algorithm on the GEE computing platform based on sample datasets with sufficient auxiliary data. The methodology combined seven axillary data and took the fast processing and variable controlling ability of both GEE and RF classification algorithms. Although the landscape of the study area is so complex and hence hinders accurate identification of pixels of different land covers, the classification accuracies and visual inspection of classified images ensured that the RF classifier yielded consistent and accurate maps for the study area.

The study demonstrates that the arid and semiarid land of the upper Tekeze basin in Waghimra Administrative Zone has experienced land use/land cover change over the last thirty-one years. The finding revealed that the water body has expanded 4.8 times from its 1990 coverage by increasing on average 81.17 ha every year 1900 to 2021. The expansion of the water body was primarily due to the accumulation of water behind the Tekeze hydroelectric dam. Forest increased by 7.36% in the first 10-year interval and decreased by 17.37 and 0.98% in the next two decades from 2000 to 2021. Bushland has shown continuous increment during the first decade (from 1990 to 2000) by about 30.74% and decreased by 9.64 and 36.34% from 2000 to 2010 and 2010 to 2021, respectively. The study revealed that most of vegetation degradation was happened during from 2000 to 2021 despite the installation of conservation and restoration government policies. Positive vegetation changes were limited to small pocket area exclosures and managed watersheds. Overall, throughout the whole study period, 2435.04, 10647.44, 3825.67 44, and 444.27 ha net gain was recorded on water bodies, shrubland, dry riverbeds, and stream channels, respectively. The water body has per year from 1990 to 2021. Dry riverbeds and stream channels and farmland have increased by 82.14 and 8.64%, respectively, during the same period. On the other hand, 8028.69, 6056.25, 3043.15, and 265.12 ha loss was recorded on bushland, bareland, shrubland, and forest cover, respectively.

The study area's Woynadega and Dega agroecological districts are better regarding vegetation cover. Furthermore, barelands and dry riverbeds are less available in these agroecological zones. The finding showed that this part of the agroecological zone has greater area coverage of bareland, riverbeds, and stream channels. However, the semiarid and arid low land part of the study area was found to be more vulnerable to land degradation because of its LULC status. Combined with a steep slope and ridged topography, the majority of the land is vulnerable to land degradation, particularly soil erosion. For instance, the lowland (Kolla agroecology) part of the study area has shown remarkable degradation expressed by the increment of bareland, riverbeds, and stream channels. Furthermore, the areal coverage of forest and bushland is minimal compared with the Dega and Weyna-dega agroecology and the upper part of the basin.

The general trend observed in the study area is the decline of forest cover, bushland, and shrubs and an increase in farmland and rural settlement, dry riverbeds and stream channels, and urban settlement. Forestland was and is very small in area coverage; no afforestation or reforestation through plantation was observed over the study period. Shrubland in the basin is characterised as sparse, short shrubs over a sandy, rocky landscape. Coupled with steep slopes, rigid topography, and high surface aridity, the observed LULC change aggravates soil erosion, biodiversity loss, disturbance of hydroclimatological balance in particular and land degradation in general in the study area.

Results from this study provide an important input to decision makers in their efforts towards sustainable land use planning and management. The study highlights the need for implementation of sustainable LULC practices such as large-scale reforestation, area exclosure, and prevention of bareland through restoration of degraded areas, conservation of forest and bushland, and limiting the expansion of cultivation areas in high slope regions. Despite being one of the most severe hotspots of land degradation due to its history of significant degradation and drought, the land restoration programs initiated decades ago have not significantly influenced the LULC dynamics. Therefore, it is crucial to evaluate the past restoration approaches and adopt new policy frameworks towards sustainable land management. In general, the study suggests that urgent measures must be taken to mitigate the observed land degradation by avoiding the future conversion of forest, bushland, and shrubland to farmland while scaling up sustainable land management, reforestation, and afforestation practices on degraded and vulnerable areas.

## Figures and Tables

**Table 1:** Details of satellite images and other datasets used in the study.

Satellite	Sensor	Date of accusation	Spatial resolution (m)
Landsat 5 SR	TM	1990-01-07	30
Landsat 5 SR	TM	2000-01-01 to 2000-02-28	30
Landsat 5 SR	TM	2010-01-01 to 2010-02-28	30
Landsat 8 SR	OLI	2021-01-28	30
ASTER GDEM			30

**Table 2:** Description of LULC classes used in the study area.

No	LULC classes	Description
1	W	An area of land covered with surface water such as lakes, rivers, and ponds
2	F	Areas covered by dense natural trees forming closed or nearly closed canopies, mainly growing naturally in the reserved land, church compounds and riverbanks, and plantation
3	BL	Areas covered with small trees, woody bushes, sparse canopy trees, dense vegetation grown in protected areas and hillsides
4	ShL	Areas covered with short shrubs and bushes with little useful wood, usually stony with a very rugged microrelief. It may include a mix of small clusters of plants or single plants dispersed on open grassland
5	FrL	Cultivated land, crop fields, fallow lands, rural settlements fenced with trees that are commonly found around homesteads
6	BrL	Areas of sand, rock or soil with very sparse to no vegetation
7	RbSc	Dry river beds, stream channels, gullies, and sandy flooded area
8	UrS	Urban settlement and residential areas

*Note.* W = water body, F = forest, BL = bushland, ShL = shrubland, FrL = farmland, BrL = bareland, RbSc = dry river beds and stream channels, and UrS = urban settlements and residential areas.

**Table 3:** Accuracy of the RF classification algorithm.

Year	Accuracy	LULC classes								OA	Kappa
		W	F	BL	ShL	FrL	BrL	RbSc	UrS		
1990	UA	0.82	0.88	0.63	0.81	0.83	0.80	0.93	1	0.83	0.75
	PA	0.95	0.82	0.71	0.8	0.91	0.9	0.45	0.005		

2000	UA	0.83	0.64	0.82	0.85	0.88	0.80	0.94	1	0.86	0.79
	PA	0.93	0.84	0.81	0.82	0.89	0.86	0.89	0.70		

2010	UA	1	0.88	0.78	0.88	0.90	0.81	0.90	1	0.88	0.83
	PA	0.97	0.77	0.82	0.82	0.93	0.92	0.76	0.75		

2021	UA	1	0.74	0.82	0.84	0.91	0.86	0.91	1	0.88	0.82
	PA	0.93	0.81	0.85	0.86	0.90	0.91	0.89	0.82		

*Note.* UA = user's accuracy, PA = producer's accuracy, OA = overall accuracy, W = water body, F = forest, BL = bushland, ShL = shrubland, FrL = farmland, BrL = bareland, RbSc = dry river beds and stream channels, and UrS = urban settlements and residential areas.

**Table 4:** Magnitude and pattern of LULC change in the upper Tekeze basin (1990–2021).

LULC classes	1990		2000		2010		2021		1990–2021 change	Annual rate of change
	(ha)	(%)	(ha)	(%)	(ha)	(%)	(ha)	(%)	(%)	(ha)
W	503.14	0.11	594.1	0.13	2123.13	0.46	2944	0.64	483.97	81.17
F	2178.8	0.47	2339	0.51	1932.7	0.42	1914	0.42	−12.17	−8.84
BL	32380.7	7.05	42333.6	9.22	38251.10	8.33	24352	5.30	−24.79	−267.62
ShL	256865.8	55.93	242284.30	52.76	235593.5	51.30	253823	55.27	−1.18	−101.44
FrL	123249.7	26.84	132724.3	28.90	137936.4	30.04	133897.2	29.16	8.64	354.91
BrL	39407.8	8.58	32421.7	7.06	34436.2	7.50	33353	7.26	−15.37	−201.87
RbSc	4657.4	1.01	6362.8	1.39	8727.13	1.90	8483	1.85	82.14	127.52
UrS	1.7	0.00	185.2	0.04	245.15	0.05	446	0.10	26778.47	14.81
Total	459245		459245		459245		459212^*∗*^			

^*∗*^No data on 33 ha.

## Data Availability

Data will be available on request.
